# Effects of Sodium-Glucose Cotransporter 2 Inhibitors on Renal Outcomes in Patients with Type 2 Diabetes: A Systematic Review and Meta-Analysis of Randomized Controlled Trials

**DOI:** 10.1038/s41598-019-49525-y

**Published:** 2019-09-10

**Authors:** Jae Hyun Bae, Eun-Gee Park, Sunhee Kim, Sin Gon Kim, Seokyung Hahn, Nam Hoon Kim

**Affiliations:** 1 Department of Internal Medicine, Korea University College of Medicine, Seoul, Republic of Korea; 2 Interdisciplinary Program in Medical Informatics, Seoul National University College of Medicine, Seoul, Republic of Korea; 3 Division of Medical Statistics, Medical Research Collaborating Center, Seoul National University Hospital, Seoul, Republic of Korea; 4 Department of Medicine, Seoul National University College of Medicine, Seoul, Republic of Korea

**Keywords:** Diabetes complications, Type 2 diabetes

## Abstract

This study was conducted to investigate the effects of sodium-glucose cotransporter 2 (SGLT2) inhibitors on individual renal outcomes in patients with type 2 diabetes. We searched MEDLINE, Embase, and the Cochrane Central Register of Controlled Trials from inception to September 2017 to identify randomized controlled trials comparing SGLT2 inhibitors with placebo or antidiabetic drugs and reporting any renal outcomes in patients with type 2 diabetes. Additionally, we identified 4 articles which were published after the predefined period to include relevant data. A meta-analysis was performed to calculate weighted mean differences (WMDs) and relative risks (RRs) with 95% confidence intervals (CIs) for each renal outcome. We included 48 studies involving 58,165 patients in the analysis. SGLT2 inhibitors significantly lowered urine albumin-to-creatinine ratio (UACR) (WMD, −14.64 mg/g; 95% CI, −25.15 to −4.12; *P* = 0.006) compared with controls. The UACR-lowering effects of SGLT2 inhibitors were greater with a higher baseline UACR. Overall changes in estimated glomerular filtration rate (eGFR) were comparable between two groups (WMD, 0.19 mL/min/1.73 m^2^; 95% CI, −0.44 to 0.82; *P* = 0.552). However, SGLT2 inhibitors significantly slowed eGFR decline in patients with a higher baseline eGFR and a longer duration of treatment. Compared with controls, SGLT2 inhibitors significantly reduced the risk of microalbuminuria (RR, 0.69; 95% CI, 0.49 to 0.97; *P* = 0.032), macroalbuminuria (RR, 0.49; 95% CI, 0.33 to 0.73; *P* < 0.001), and worsening nephropathy (RR, 0.73; 95% CI, 0.58 to 0.93; *P* = 0.012). In addition, the risk of end-stage renal disease was significantly lower in SGLT2 inhibitors than in controls (RR, 0.70; 95% CI, 0.57 to 0.87; *P* = 0.001). In conclusion, SGLT2 inhibitors had beneficial renal effects by lowering the risk of albuminuria development or progression and reducing the risk of end-stage renal disease compared with placebo or other antidiabetic drugs.

## Introduction

Sodium-glucose cotransporter 2 (SGLT2) inhibitors are a novel class of antidiabetic agents which lower blood glucose levels mainly by reducing glucose reabsorption in the renal proximal tubule, leading to an increase in urinary glucose and sodium excretion^1–3^. As a result of increased glycosuria and natriuresis, the beneficial effects of SGLT2 inhibitors extend beyond glycemic control to reducing intraglomerular hypertension, promoting plasma volume contraction, lowering blood pressure (BP), reducing body weight, and decreasing uric acid levels^4,5^. Given their intrarenal and extrarenal effects, SGLT2 inhibitors have been suggested to confer renoprotection in patients with type 2 diabetes.

Several studies have reported beneficial effects of SGLT2 inhibitors on the kidney^6–10^. In large clinical trials, empagliflozin and canagliflozin similarly reduced the risk of progression of albuminuria and the composite of sustained decrease in renal function, renal replacement therapy (RRT), or renal death compared with placebo^6,9^. Recently, dapagliflozin also reduced the composite renal outcome of 40% decrease in estimated glomerular filtration rate (eGFR), end-stage renal disease (ESRD), or renal death compared with placebo^11^. However, these studies were performed in patients with an established cardiovascular disease or high cardiovascular risk^9,11,12^. Therefore, it is difficult to conclude that the beneficial renal effects of SGLT2 inhibitors extend to overall patients, especially with low cardiovascular risk. Furthermore, individual renal outcomes of these studies have been inconsistent. In the Empagliflozin Cardiovascular Outcome Event Trial in Type 2 Diabetes Mellitus Patients (EMPA-REG OUTCOME) trial, empagliflozin significantly lowered the risk of initiation of RRT but did not affect new-onset microalbuminuria^6^. In the Canagliflozin Cardiovascular Assessment Study (CANVAS) Program, canagliflozin significantly reduced the risk of new-onset microalbuminuria and 40% decrease in eGFR with no difference in the need for RRT^9,13^. In both studies^6,9^, renal outcomes were secondary endpoints and the number of events indicating ESRD was not sufficient to provide conclusive information. Moreover, the Dapagliflozin Effect on Cardiovascular Events-Thrombolysis in Myocardial Infarction 58 (DECLARE-TIMI 58) trial did not reported individual renal outcomes^11^. Consequently, the renoprotective effects of SGLT2 inhibitors need to be elucidated by more convincing evidence.

Concerns have also been raised over the adverse renal events in patients with SGLT2 inhibitor treatment because these agents are associated with a decrease in intravascular volume and an acute decline in eGFR^14–16^. Canagliflozin and dapagliflozin have increased the risk of acute kidney injury in patients who have predisposing factors including hypoglycemia, chronic kidney disease (CKD), heart failure, and potentially nephrotoxic drugs^14,17,18^.

In this regard, we conducted this systematic review and meta-analysis of randomized controlled trials (RCTs) to investigate the effects of SGLT2 inhibitors on individual renal outcomes compared with placebo or other antidiabetic drugs in patients with type 2 diabetes.

## Methods

### Study design

This systematic review and meta-analysis was performed based on a prespecified protocol developed by the authors (Supplementary Appendix 1), and the results were reported according to the Preferred Reporting Items for Systematic Reviews and Meta-Analyses (PRISMA) statement (Supplementary Appendix 2)^19^.

### Data sources and search strategy

We searched MEDLINE, Embase, and the Cochrane Central Register of Controlled Trials to identify RCTs of SGLT2 inhibitors with full-text articles published from inception to September 2017, regardless of language and publication status. The search terms used for SGLT2 inhibitors were SGLT2 inhibitor or SGLT-2 inhibitor or canagliflozin or dapagliflozin or empagliflozin or ertugliflozin or ipragliflozin or luseogliflozin or remogliflozin or sergliflozin or tofogliflozin (Supplementary Appendix 3). In addition, we identified 4 articles^11,20–22^ which were published after the predefined period to include all relevant data.

### Study selection

The RCTs comparing SGLT2 inhibitors with placebo or other antidiabetic drugs with ≥ 12 weeks of study duration in type 2 diabetes were included. We eliminated duplicate publications of original RCT and screened titles and abstracts. Among them, we selected RCTs that reported at least one of the following renal outcomes: changes in urine albumin-to-creatinine ratio (UACR) or eGFR, and incident microalbuminuria, macroalbuminuria, doubling of serum creatinine, renal failure, ESRD, RRT, dialysis, or kidney transplantation. The selection criteria for renal outcomes have been reported elsewhere^23^. Pooled analyses or secondary analyses were included when they provided additional information about renal outcomes beyond that found in original RCT articles. We included two publications of canagliflozin trials^20,22^ which were reported after the prespecified analysis considering the effect size and weight of these studies.

### Data extraction

Two authors (J.H.B. and E.P.) independently extracted data according to the prespecified protocol. The procedure of extracting data from publications have been reported elsewhere^23^. Briefly, the renal outcomes of interests were changes in UACR and eGFR, incident microalbuminuria (UACR >30 mg/g) and macroalbuminuria (UACR >300 mg/g), worsening nephropathy (defined as development of microalbuminuria or macroalbuminuria from normoalbuminuria, or progression from microalbuminuria to macroalbuminuria), and the development of ESRD. We extracted mean changes from baseline and their standard deviations for continuous variables in the intervention (SGLT2 inhibitors) and control (placebo or other antidiabetic drugs) groups and used them as the summary measures. The number of patients reporting each renal outcome was extracted for dichotomous variables. We also collected information about the first author, publication year, number and mean age of randomized participants, study duration, intervention and comparison treatment, background therapy for glycemic control, baseline UACR and eGFR, and the history of cardiovascular disease, heart failure, and CKD (eGFR <60 mL/min/1.73 m^2^).

### Quality assessment

We used the Cochrane Risk of Bias Tool to assess quality and risk of bias for included studies^24^. Two authors (J.H.B. and E.P.) independently reviewed each RCT and classified the risk of bias as adequate (low risk of bias), unclear (unclear risk of bias), or inadequate (high risk of bias) based on six aspects of trials: sequence generation, allocation concealment, blinding of participants and personnel, incomplete outcome data, selective reporting, and other sources of bias^24^. Any disagreements were resolved by consensus among the authors (J.H.B., N.H.K., and S.H.).

### Data synthesis and analysis

We calculated weighted mean differences (WMDs) with 95% confidence intervals (CIs) to assess effect size for continuous variables including UACR and eGFR. We also calculated the relative risks (RRs) with 95% CIs to estimate effect size for dichotomous variables including microalbuminuria, macroalbuminuria, worsening nephropathy, and ESRD. In the meta-analysis, we used a random effects model to combine estimators. We also considered a fixed effect model additionally for exploration of the discrepancy in results. The *I*^2^ statistic, *τ*^2^ statistic, and Cochran’s Q test were used to assess statistical heterogeneity among the studies^25^. We regarded the *I*^2^ statistic of 0% to 40%, 30% to 60%, 50% to 90%, and 75% to 100% as not significant, moderate, substantial, and considerable heterogeneity, respectively^26^. To detect reporting bias, such as publication bias, asymmetry in the funnel plot was evaluated for renal outcomes^27,28^. We performed a subgroup analysis of eGFR based on baseline eGFR (<60, 60–90, and >90 mL/min/1.73 m^2^) and study duration (<26, 26–52, and >52 weeks), and sensitivity analyses of microalbuminuria, macroalbuminuria, worsening nephropathy, and ESRD by considering only canagliflozin, dapagliflozin, and empagliflozin for SGLT2 inhibitors. Additionally, prespecified meta-regression was conducted for changes in UACR according to baseline UACR and for changes in eGFR according to baseline eGFR and study duration, respectively. Some outliers were eliminated by diagnostic measures^29^. All statistical analyses were performed using R version 3.1.0 (R Foundation for Statistical Computing, Vienna, Austria). *P* values of < 0.05 and <0.10 were regarded as statistically significant for treatment effects and test for heterogeneity, respectively.

## Results

### Characteristics of included studies

**Figure 1:**
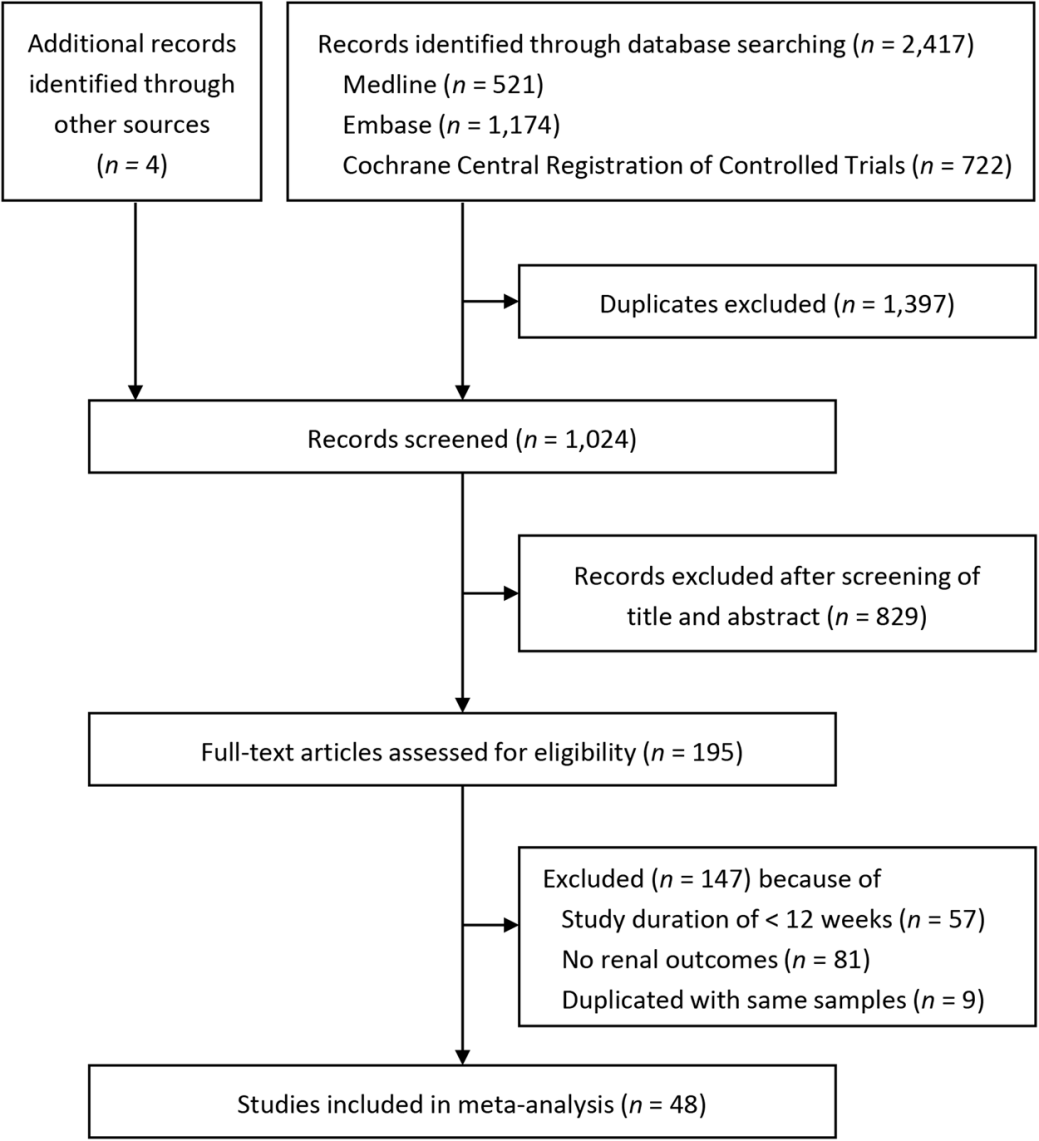
Study screening and selection process.

The study screening and selection process is shown in Fig. 1. Of 2,421 records retrieved through the database search, 48 studies were included in the systematic review and meta-analysis. Two of the 48 studies^7,30^ were pooled analyses of five RCTs^31–35^ and another five RCTs^15,36–39^. The characteristics of the included studies and their participants are described in Supplementary Table 1. The total number of participants was 58,165 (34,661 in the SGLT2 inhibitor group and 23,504 in the control group). The number of participants in each study ranged from 114 to 10,142. Three studies had a duration of 187 to 296 weeks^6,9,22^, whereas the remaining studies had a duration ranging from 12 to 104 weeks. The baseline eGFR of the participants was ≥55 (or 60) mL/min/1.73 m^2^ in 24 studies^4,8,38–59^, ≥30 mL/min/1.73 m^2^ in 14 studies^6,7,9,15,20,22,30,32–34,60–63^, ≥20 mL/min/1.73 m^2^ and in 1 study^21^. In one study, 74 of 741 participants had an eGFR of ≥15 and <30 mL/min/1.73 m^2^ at baseline^35^.

### Assessment of study quality and risk of bias

The risk of bias assessment is summarized in Supplementary Fig. 1. Forty-four of the 48 studies reported adequate random sequence generation and adequate allocation concealment. Three studies did not describe the method of sequence generation and allocation concealment^15,63,64^. All 48 studies described adequate blinding of participants and personnel. Moreover, 14 studies reported incomplete outcome data due to losses to follow-up^15,30,42,46,48–51,58,60–62,64,65^. One study had the possibility of selective reporting in the dapagliflozin 5 mg group^40^.

### Changes in UACR and eGFR

**Figure 2:**
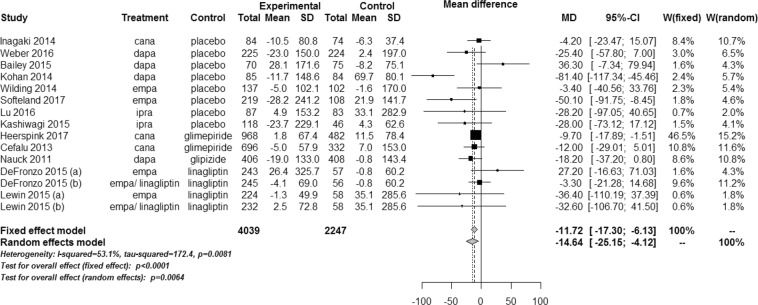
Weighted mean differences in changes in urine albumin-to-creatinine ratio from baseline (mg/g) for sodium-glucose cotransporter 2 inhibitors versus placebo or other antidiabetic drugs. CI, confidence interval; MD, mean difference; SD, standard deviation; W, weight.

**Figure 3:**
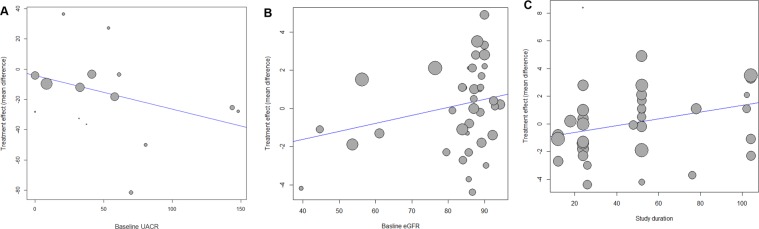
Meta-regression of changes in urine albumin-to-creatinine ratio (UACR) and estimated glomerular filtration rate (eGFR) for sodium-glucose cotransporter 2 inhibitors versus placebo or other antidiabetic drugs. (**A**) Changes in UACR according to baseline UACR. (**B**) Changes in eGFR according to baseline eGFR. (**C**) Changes in eGFR according to treatment duration.

**Figure 4:**
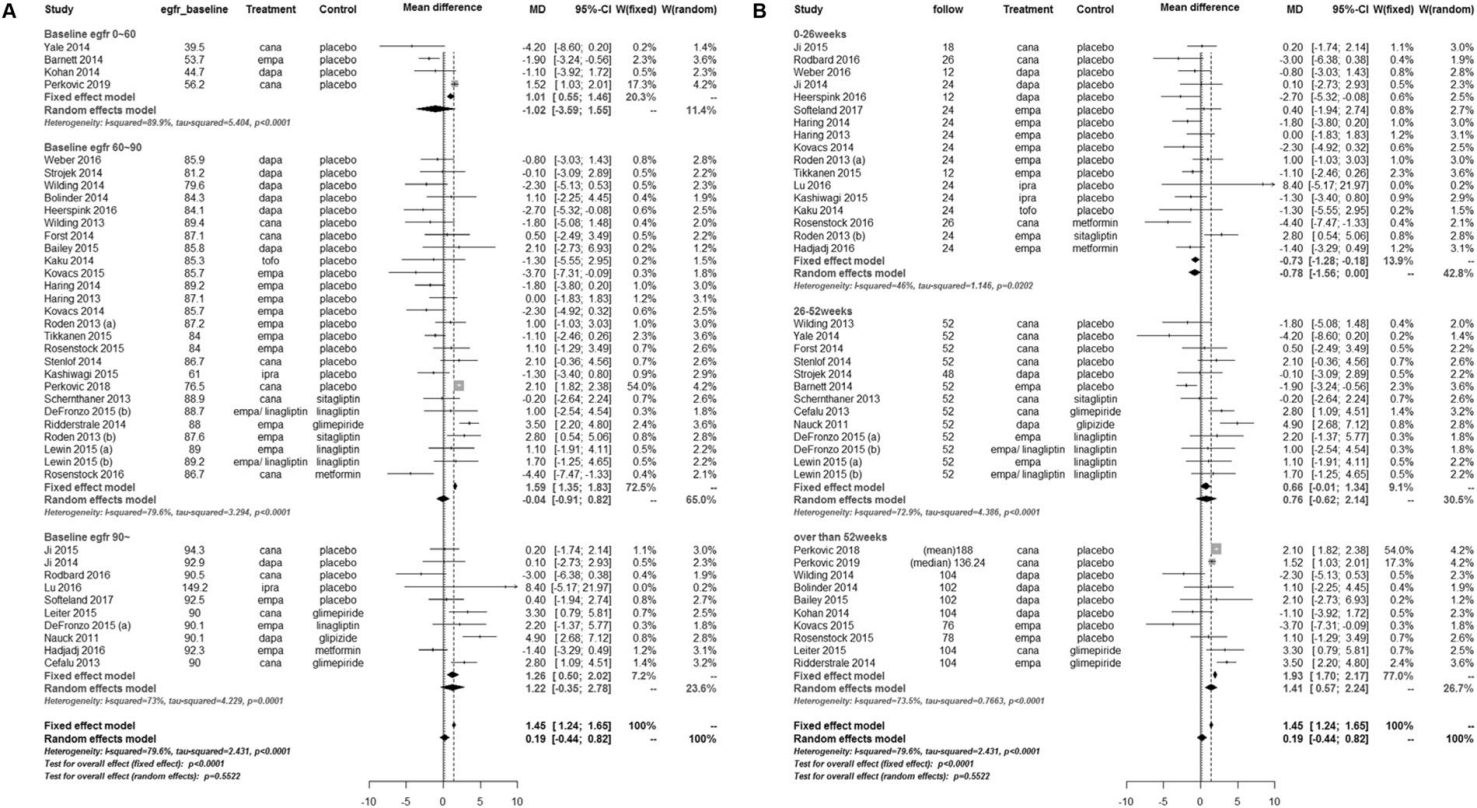
Weighted mean differences in estimated glomerular filtration rate from baseline (mL/min/1.73 m^2^) for sodium-glucose cotransporter 2 inhibitors versus placebo or other antidiabetic drugs. (**A**) According to baseline estimated glomerular filtration rate. (**B**) According to treatment duration. CI, confidence interval; MD, mean difference; SD, standard deviation; W, weight.

SGLT2 inhibitors significantly lowered the UACR compared with controls (WMD, −14.64 mg/g; 95% CI, −25.15 to −4.12; *P* = 0.006) (Fig. 2). The test for heterogeneity showed moderate heterogeneity across the studies (*I*^2^ = 53.1%; *P* = 0.008). In the meta-regression, the UACR-lowering effects of SGLT2 inhibitors tended to be greater with higher levels of baseline UACR (*P* = 0.081) (Fig. 3A). The changes in eGFR were not significantly different between SGLT2 inhibitors and controls (WMD, 0.19 mL/min/1.73 m^2^; 95% CI, −0.44 to 0.82; *P* = 0.552) (Fig. 4A,B). The test for heterogeneity for this showed substantial heterogeneity across the studies (*I*^2^ = 79.6%; *P* < 0.001). There was a large discrepancy noted in estimated treatment effects between fixed effect and random effects models, depending on weights given to two large trials^20,22^. However, SGLT2 inhibitors significantly slowed the decline in eGFR in patients with >52 weeks of treatment duration compared with controls (Fig. 4B). In the meta-regression, the decline in eGFR were slower in patients with a higher baseline eGFR (*P* = 0.116) (Fig. 3B) and a longer duration of follow-up (*P* = 0.038) (Fig. 3C).

### Development or progression of albuminuria

**Figure 5:**
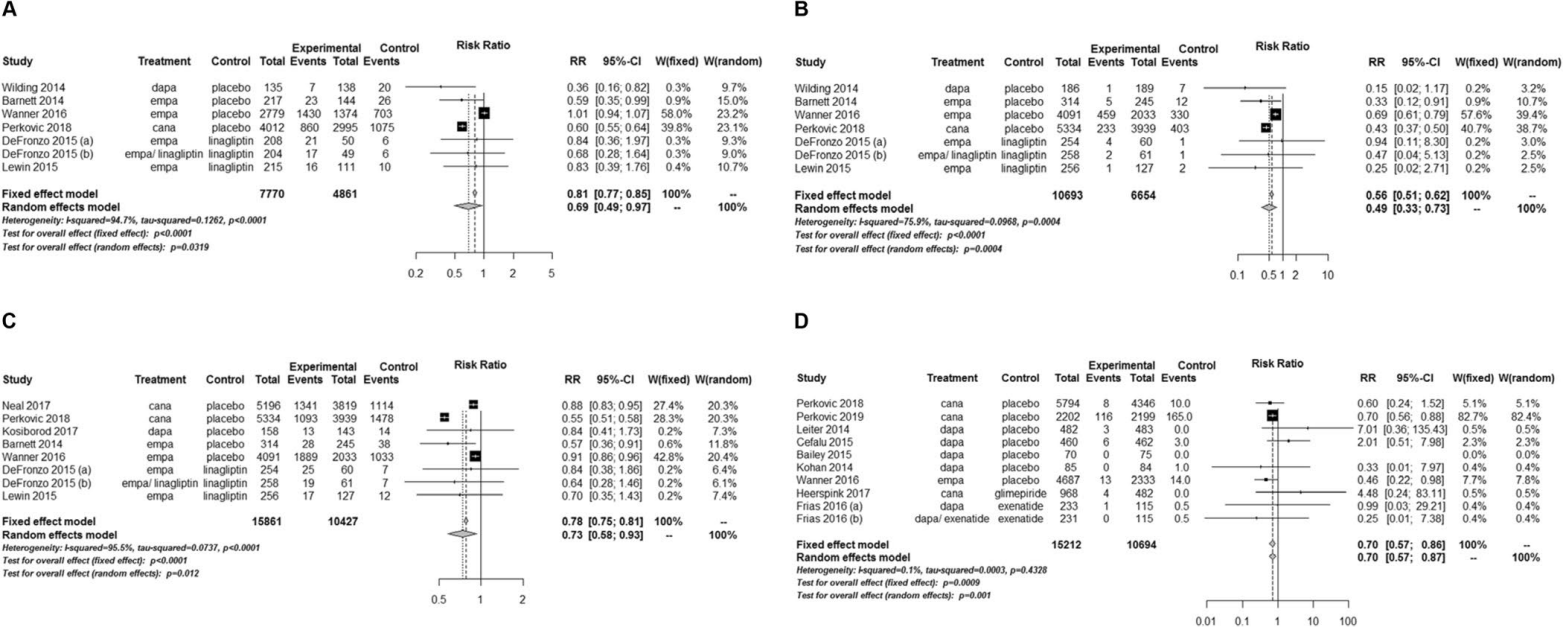
Relative risks of microalbuminuria, macroalbuminuria, worsening nephropathy, and end-stage renal disease for sodium-glucose cotransporter 2 inhibitors versus placebo or other antidiabetic drugs. (**A**) Microalbuminuria. (**B**) Macroalbuminuria. (**C**) Worsening nephropathy. (**D**) End-stage renal disease. CI, confidence interval; RR, relative risk; W, weight.

SGLT2 inhibitors significantly lower the risk of developing microalbuminuria compared with controls (RR, 0.69; 95% CI, 0.49 to 0.97; *P* = 0.032) (Fig. 5A). The beneficial effects on microalbuminuria were primarily driven by one large trial with canagliflozin^22^. The test for heterogeneity showed considerable heterogeneity across the studies (*I*^2^ = 94.7%; *P* < 0.001). SGLT2 inhibitors significantly lowered the risk of developing macroalbuminuria compared with controls (RR, 0.49; 95% CI, 0.33 to 0.73; *P* < 0.001) (Fig. 5B). There was substantial heterogeneity across the studies for this (*I*^2^ = 75.9%; *P* < 0.001). In addition, SGLT2 inhibitors significantly lowered the risk of worsening nephropathy compared with controls (RR, 0.73; 95% CI, 0.58 to 0.93; *P* = 0.012) (Fig. 5C). There was considerable heterogeneity across the studies (*I*^2^ = 95.5%; *P* < 0.001). In subgroup analyses, canagliflozin reduced both the risk of microalbuminuria and macroalbuminuria. On the other hand, dapagliflozin and empagliflozin reduce the risk of microalbuminuria and macroalbuminuria, respectively (Supplementary Fig. 2A–C).

### Development of ESRD

SGLT2 inhibitors significantly reduced the risk of ESRD compared with controls (RR, 0.70; 95% CI, 0.57 to 0.87; *P* = 0.001) (Fig. 5D). The number of events was 151 of 15,212 and 194 of 10,694 participants in the SGLT2 inhibitor and control groups, respectively. Heterogeneity was regarded as not significant across the studies (*I*^2^ = 0.1%; *P* = 0.433).

### Assessment of funnel plot asymmetry

In the changes in UACR and eGFR, the funnel plots did not show any notable asymmetry apart from a few outlying values (Supplementary Fig. 3A,B). Although it was hard to determine asymmetry of the plots for in incident microalbuminuria, incident macroalbuminuria, and worsening nephropathy (Supplementary Fig. 4A–C) due to the small number of studies, the funnel plot still appeared to be quite symmetric in the development of ESRD (Supplementary Fig. 4D).

## Discussion

This systematic review and meta-analysis found that SGLT2 inhibitors were associated with a significantly lower risk of development or progression of albuminuria compared with placebo or other antidiabetic drugs in patients with type 2 diabetes. The UACR-lowering effects of SGLT2 inhibitors were associated with a higher baseline UACR. The overall changes in eGFR were not different between two groups. However, SGLT2 inhibitors slowed the decline in eGFR in patients with a higher baseline eGFR and a longer duration of treatment. In addition, SGLT2 inhibitor significantly reduced the risk of ESRD compared with controls.

Considering the direct action of SGLT2 inhibitors on the renal tubules and their favorable effects on BP, body weight, and heart failure, these agents have been suggested theoretically to improve renal outcomes in patients with type 2 diabetes^5,66^. The large clinical trials already showed improvement in the composite renal outcomes with SGLT2 inhibitors^6,9,11^. However, these studies were conducted in patients with an average age of 60 years, a long diabetes duration of about 10 years, and an established cardiovascular disease or high cardiovascular risk. In the present study, we demonstrated that SGLT2 inhibitors had the renoprotective effects by reducing the risk of albuminuria and ESRD in patients with a wide range of cardiovascular risk.

SGLT2 inhibitors may reduce albuminuria by several mechanisms including a decrease in glomerular hyperfiltration^67^, improvement in tubulointerstitial fibrosis^68^, systemic BP reduction^69^, changes in plasma volume expansion^70^, and a decrease in uric acid levels^71^. In patients with type 2 diabetes and either microalbuminuria or macroalbuminuria, empagliflozin reduced the UACR independent of changes in hemoglobin A1c (HbA_1c_), BP, and body weight^7^. Dapagliflozin also reduced the UACR for over 2 years of treatment in patients with type 2 diabetes and stage 3 CKD regardless of changes in HbA_1c_, BP, eGFR, and uric acid^72^. These findings suggest that SGLT2 inhibitors reduce albuminuria through their direct effects on the kidney. In diabetic mice, SGLT2 inhibitors reduced albuminuria by ameliorating intraglomerular hypertension and tubulointerstitial fibrosis^73,74^, which are the two key contributors to renal damage in diabetic kidney disease (DKD). In line with these findings, our meta-analysis showed that albuminuria-lowering effects of SGLT2 inhibitors were higher on macroalbuminuria than on microalbuminuria. It could be partly explained by the greater UACR reduction in patients with a higher baseline UACR after SGLT2 inhibitor treatment. Therefore, SGLT2 inhibitors may have beneficial effects on albuminuria in the later stage rather than the early stage of DKD, which needs to be evaluated in further studies.

The overall changes in eGFR showed no difference between SGLT2 inhibitors and controls. In the subgroup-analysis and meta-regression, we found that changes in renal function were affected by baseline eGFR and duration of treatment. The changes in renal function after SGLT2 inhibitor treatment were characterized by a rapid decline in eGFR within the first 4–5 weeks, followed by progressive recovery over time^15,35,50^. In addition, the decrease in eGFR was reversible within 2 weeks after drug discontinuation^35^. These findings indicate that the changes in eGFR with SGLT2 inhibitor treatment were a consequence of the drug’s hemodynamic effects. In patients with type 1 diabetes, empagliflozin attenuated renal hyperfiltration accompanied by a decrease in eGFR through affecting tubuloglomerular feedback^67^. Reduction in renal hyperfiltration may be beneficial against progressive decline in renal function because intraglomerular hyperfiltration increases the risk of development and progression of DKD^75–77^. Besides intrarenal effects, SGLT2 inhibitors may affect eGFR by reducing BP and body weight. In two recent trials, dapagliflozin and empagliflozin similarly maintained BP and weight reduction despite a decline in renal function, whereas HbA_1c_ reduction was decreased^78,79^, suggesting that they may slow the progressive decline in renal function independent of their glucose-lowering effects^8^.

Finally, SGLT2 inhibitors significantly reduced the risk of ESRD compared with placebo or other antidiabetic drugs. The direction of treatment effects of dapagliflozin was different from those of canagliflozin and empagliflozin but it did not change the overall treatment effect. Considering their beneficial effects on albuminuria, progressive eGFR decline, and glomerular hyperfiltration^4^, SGLT2 inhibitors have been expected to improve hard renal outcomes. In the Canagliflozin and Renal Endpoints in Diabetes with Established Nephropathy Clinical Evaluation (CREDENCE) trial, which evaluated the primary composite renal outcome, canagliflozin showed 30% risk reduction of the composite of ESRD, doubling of serum creatinine, or renal or cardiovascular death, and 32% of risk reduction of ESRD compared with placebo in patients with type 2 diabetes and albuminuric CKD^22^. All the patients in this study were receiving angiotensin-converting-enzyme inhibitor or angiotensin-receptor blocker^22^. Interestingly, the magnitude of renal benefit of SGLT2 inhibitors was greater in patients with less severe kidney disease at baseline in a meta-analysis of cardiovascular outcome trials^80^. Therefore, further investigation is required to determine whether the risk reduction of ESRD is a class effect of SGLT2 inhibitors in patients with or without CKD.

Our study has some limitations. First, most of the studies included in our meta-analysis were originally designed to investigate glucose-lowering effects and safety of SGLT2 inhibitors. Therefore, the analysis of renal outcomes should be interpreted cautiously. Second, 14 of 48 studies reported incomplete outcome data due to losses to follow-up, suggesting the possibility of attrition bias. Third, we could not evaluate the renal effects of SGLT2 inhibitors stratified by cardiovascular risk of included studies because they provided missing or inconsistent data. Recently, another meta-analysis showed that SGLT2 inhibitors reduced the risk of the composite of worsening of renal function, ESRD, or renal death similarly in patients with or without atherosclerotic cardiovascular disease^80^.

In conclusion, our meta-analysis demonstrated that SGLT2 inhibitors had beneficial effects on the kidney by lowering the risk of albuminuria development or progression and reducing the risk of ESRD compared with placebo or other antidiabetic drugs in patients with type 2 diabetes. In addition, the renoprotective effects of SGLT2 inhibitors were greater in patients with a higher UACR and GFR, and a long duration of treatment.

## Supplementary information

Supplementary Information
