# Supplementary material for: Effects of Sodium-Glucose Cotransporter 2 Inhibitors on Renal Outcomes in Patients with Type 2 Diabetes: A Systematic Review and Meta-Analysis of Randomized Controlled Trials
Source: Sci Rep. 2019 Sep 10;9:13009. doi: 10.1038/s41598-019-49525-y (PMC6736944; doi:10.1038/s41598-019-49525-y)
Supplement: Supplementary file 1 — Supplementary Information [file 41598_2019_49525_MOESM1_ESM.docx]

**Effects of** **Sodium-Glucose Cotransporter 2 Inhibitors on Renal Outcomes in Patients with Type 2 Diabetes: A Systematic Review and Meta-Analysis of Randomized Controlled Trials**

Jae Hyun Bae, Eun-Gee Park, Sunhee Kim, Sin Gon Kim, Seokyung Hahn, Nam Hoon Kim

**Supplementary Information**

**Supplementary Appendix 1.** Study protocol

1. **Title:** Effects of sodium-glucose cotransporter 2 inhibitors on renal outcomes in patients with type 2 diabetes: a systematic review and meta-analysis of randomized controlled trials
2. **Objectives:** This study aimed to investigate the effects of sodium-glucose cotransporter 2 (SGLT2) inhibitors on renal outcomes in patients with type 2 diabetes compared with placebo or other antidiabetic drugs.
3. **Protocol and registration:** Methods of database search, study selection, data extraction, assessment of study quality and risk of bias, and statistical analysis were prespecified in the protocol at the beginning of the study.
4. **Reporting:** This systematic review and meta-analysis was reported according to the Preferred Reporting Items for Systematic Reviews and Meta-Analyses (PRISMA) statement.
5. **Eligibility criteria**
   1. Study characteristics
      1. Population: patients with type 2 diabetes
      2. Intervention: SGLT2 inhibitors
      3. Comparison: placebo or other antidiabetic drugs
      4. Outcomes of interests
         1. Changes in urine albumin-to-creatinine ratio (UACR)
         2. Changes in estimated glomerular filtration rate (eGFR)
         3. Development of microalbuminuria
         4. Development of macroalbuminuria
         5. Worsening nephropathy (defined as development of microalbuminuria or macroalbuminuria from normoalbuminuria, or progression from microalbuminuria to macroalbuminuria)
         6. Development of end-stage renal disease (ESRD) (defined as initiation of renal replacement therapy, renal transplantation, or renal failure)
      5. Study design: randomized controlled trials (RCTs)
      6. Length of follow-up: at least 12 weeks of study duration
   2. Report characteristics
      1. Years considered: published until September 2017
      2. Language: no limitation for language
      3. Publication status: full-text articles with no limitation for publication status
   3. Inclusion and exclusion criteria
      1. The study population comprised men and women with type 2 diabetes.
      2. We only included RCTs comparing the efficacy or safety of SGLT2 inhibitors with that of placebo or other antidiabetic drugs in patients with type 2 diabetes.
      3. Any concurrent use of antidiabetic drugs was allowed including oral antidiabetic drugs, insulin, and glucagon-like peptide-1 receptor agonists other than those for intervention and comparison.
      4. Only RCTs with a study duration of ≥ 12 weeks were included.
      5. We only included RCTs reporting at least one of the following renal outcomes: UACR, eGFR, microalbuminuria, macroalbuminuria, doubling of serum creatinine, renal failure, ESRD, renal replacement therapy, dialysis, or kidney transplantation.
      6. In case of duplicates or extensions, we only included the study with the longer duration or more information about renal outcomes.
      7. Pooled analyses or secondary analyses were only included only when they provided more information about renal outcomes than original articles.
      8. There is no limitation for language.
      9. We included full-text articles with no limitation for publication status.
6. **Information sources:** We searched the electronic databases including MEDLINE, Embase, and the Cochrane Central Register of Controlled Trials.
7. **Search strategy:** RCTs of SGLT2 inhibitors in patients with type 2 diabetes were searched using the following search terms:
   1. MEDLINE: SGLT2 inhibitor OR SGLT-2 inhibitor OR canagliflozin OR dapagliflozin OR empagliflozin OR ertugliflozin OR ipragliflozin OR luseogliflozin OR remogliflozin OR sergliflozin OR tofogliflozin
   2. Embase: SGLT2 inhibitor OR SGLT-2 inhibitor OR canagliflozin OR dapagliflozin OR empagliflozin OR ertugliflozin OR ipragliflozin OR luseogliflozin OR remogliflozin OR sergliflozin OR tofogliflozin
   3. The Cochrane Central Register of Controlled Trials: SGLT2 inhibitor OR SGLT-2 inhibitor OR canagliflozin OR dapagliflozin OR empagliflozin OR ertugliflozin OR ipragliflozin OR luseogliflozin OR remogliflozin OR sergliflozin OR tofogliflozin
8. **Study selection:** All identified records were screened and evaluated for eligibility by two reviewers independently. We reviewed titles, abstracts, and full texts of the studies. Any disagreements were resolved by consensus among the investigators of this study.
9. **Data extraction:** Standardized data extraction was performed by two reviewers independently as follows. Any discrepancies were resolved by consensus among the investigators of this study.
   1. First author
   2. Publication year
   3. Number of randomized participants
   4. Mean age of randomized participants in years
   5. Study duration in weeks
   6. Intervention including names and doses of SGLT2 inhibitors
   7. Comparison including placebo or names and doses of other antidiabetic drugs
   8. Background antidiabetic drugs
   9. Baseline eGFR in mL/min/1.73 m^2^
   10. Mean changes in UACR with their standard deviations in mg/g
   11. Mean changes in eGFR with their standard deviations in mL/min/1.73 m^2^
   12. Number of participants reporting the development of microalbuminuria
   13. Number of participants reporting the development of macroalbuminuria
   14. Number of participants reporting worsening nephropathy
   15. Number of participants reporting the development of ESRD
10. **Assessment of study quality and risk bias:** We assessed the quality and risk of bias of the included studies using the Cochrane Risk of Bias Tool. Two reviewers evaluated each study based on following aspects of trials:
    1. Random sequence generation
    2. Allocation concealment
    3. Blinding
    4. Incomplete outcome data
    5. Selective reporting
    6. Other sources of bias
11. **Data synthesis**
    1. Statistical analysis: We calculated weighted mean differences (WMDs) with 95% CIs for continuous variables and relative risks (RRs) with 95% CIs for dichotomous variables. In the meta-analysis, we used a random effects model to combine estimators. We also considered a fixed effect model additionally for exploration of the discrepancy in results.
    2. Sensitivity analysis: We performed a subgroup analysis of eGFR according to baseline eGFR and study duration. In addition, we conducted prespecified meta-regression for changes in UACR according to baseline UACR and for changes in eGFR according to study duration and baseline eGFR.
    3. Identifying and measuring statistical heterogeneity: We assessed statistical heterogeneity among the studies using the *I*^2^ statistic, *τ*^2^ statistic, and Cochran’s Q test.

**Supplementary Appendix 2.** PRISMA statement

| **Section/topic** | **#** | **Checklist item** | **Reported on page #** |
| --- | --- | --- | --- |
| **TITLE** | | | |
| Title | 1 | Identify the report as a systematic review, meta-analysis, or both. | 1 |
| **ABSTRACT** | | | |
| Structured summary | 2 | Provide a structured summary including, as applicable: background; objectives; data sources; study eligibility criteria, participants, and interventions; study appraisal and synthesis methods; results; limitations; conclusions and implications of key findings; systematic review registration number. | 3 |
| **INTRODUCTION** | | | |
| Rationale | 3 | Describe the rationale for the review in the context of what is already known. | 4, 5 |
| Objectives | 4 | Provide an explicit statement of questions being addressed with reference to participants, interventions, comparisons, outcomes, and study design (PICOS). | 5, Supplementary Appendix 1 |
| **METHODS** | | | |
| Protocol and registration | 5 | Indicate if a review protocol exists, if and where it can be accessed (e.g., Web address), and, if available, provide registration information including registration number. | 5, Supplementary Appendix 1 |
| Eligibility criteria | 6 | Specify study characteristics (e.g., PICOS, length of follow-up) and report characteristics (e.g., years considered, language, publication status) used as criteria for eligibility, giving rationale. | 6,  Supplementary Appendix 1 |
| Information sources | 7 | Describe all information sources (e.g., databases with dates of coverage, contact with study authors to identify additional studies) in the search and date last searched. | 5,  Supplementary Appendix 1 |
| Search | 8 | Present full electronic search strategy for at least one database, including any limits used, such that it could be repeated. | 5, 6, Supplementary Appendix 1, 3 |
| Study selection | 9 | State the process for selecting studies (i.e., screening, eligibility, included in systematic review, and, if applicable, included in the meta-analysis). | 6 |
| Data collection process | 10 | Describe method of data extraction from reports (e.g., piloted forms, independently, in duplicate) and any processes for obtaining and confirming data from investigators. | 6, 7 |
| Data items | 11 | List and define all variables for which data were sought (e.g., PICOS, funding sources) and any assumptions and simplifications made. | 6, 7,  Supplementary Appendix 1 |
| Risk of bias in individual studies | 12 | Describe methods used for assessing risk of bias of individual studies (including specification of whether this was done at the study or outcome level), and how this information is to be used in any data synthesis. | 7 |
| Summary measures | 13 | State the principal summary measures (e.g., risk ratio, difference in means). | 7, 8 |
| Synthesis of results | 14 | Describe the methods of handling data and combining results of studies, if done, including measures of consistency (e.g., *I^2^*) for each meta-analysis. | 7, 8 |
| Risk of bias across studies | 15 | Specify any assessment of risk of bias that may affect the cumulative evidence (e.g., publication bias, selective reporting within studies). | 8 |
| Additional analyses | 16 | Describe methods of additional analyses (e.g., sensitivity or subgroup analyses, meta-regression), if done, indicating which were pre-specified. | 8 |
| **RESULTS** | | | |
| Study selection | 17 | Give numbers of studies screened, assessed for eligibility, and included in the review, with reasons for exclusions at each stage, ideally with a flow diagram. | 8, 9,  Figure 1 |
| Study characteristics | 18 | For each study, present characteristics for which data were extracted (e.g., study size, PICOS, follow-up period) and provide the citations. | 8, 9,  Supplementary Table 1 |
| Risk of bias within studies | 19 | Present data on risk of bias of each study and, if available, any outcome level assessment (see item 12). | 9, Supplementary Figure 1 |
| Results of individual studies | 20 | For all outcomes considered (benefits or harms), present, for each study: (a) simple summary data for each intervention group (b) effect estimates and confidence intervals, ideally with a forest plot. | 9, 10,  Figure 2, 4, 5 |
| Synthesis of results | 21 | Present results of each meta-analysis done, including confidence intervals and measures of consistency. | 9, 10,  Figure 2, 4, 5 |
| Risk of bias across studies | 22 | Present results of any assessment of risk of bias across studies (see Item 15). | 10, 11,  Supplementary Figure 3, 4 |
| Additional analysis | 23 | Give results of additional analyses, if done (e.g., sensitivity or subgroup analyses, meta-regression [see Item 16]). | 9, 10,  Figure 3, Supplementary Figure 2 |
| **DISCUSSION** | | | |
| Summary of evidence | 24 | Summarize the main findings including the strength of evidence for each main outcome; consider their relevance to key groups (e.g., healthcare providers, users, and policy makers). | 11 |
| Limitations | 25 | Discuss limitations at study and outcome level (e.g., risk of bias), and at review-level (e.g., incomplete retrieval of identified research, reporting bias). | 13, 14 |
| Conclusions | 26 | Provide a general interpretation of the results in the context of other evidence, and implications for future research. | 14 |
| **FUNDING** | | | |
| Funding | 27 | Describe sources of funding for the systematic review and other support (e.g., supply of data); role of funders for the systematic review. | 14 |

**Supplementary Appendix 3.** Search strategy

Search data: September 2017

| Data source | Search terms |
| --- | --- |
| MEDLINE | #1. Sodium-glucose cotransporter  #2. SGLT2 OR SGLT-2 OR SGLT 2  #3. Canagliflozin OR Invokana  #4. Dapagliflozin OR Farxiga OR Forxiga  #5. Empagliflozin OR Jardiance  #6. Ertugliflozin  #7. Ipragliflozin OR Suglat  #8. Luseogliflozin OR Lusefi  #9. Sotagliflozin OR LX4211  #10. Remogliflozin  #11. Sergliflozin  #12. Tofogliflozin OR Apleway OR Deberza  #13. OR #1-#12  #14. Random*  #15. “Randomized Controlled Trial” [Publication Type]  #16. RCT or RCTs  #17. #14 OR #16  #18. #13 AND #17 |
| CENTRAL | TITLE-ABSTRACT-KEYWORDS (Sodium-glucose cotransporter OR SGLT2 OR SGLT-2 OR SGLT 2 OR canagliflozin OR dapagliflozin OR empagliflozin OR ertugliflozin OR ipragliflozin OR luseogliflozin OR sotagliflozin OR remogliflozin OR sergliflozin OR tofogliflozin) |
| Embase | (TITLE-ABSTRACT-INDEX TERM (Sodium-glucose cotransporter OR SGLT2 OR SGLT-2 OR SGLT 2 OR canagliflozin OR dapagliflozin OR empagliflozin OR ertugliflozin OR ipragliflozin OR luseogliflozin OR sotagliflozin OR remogliflozin OR sergliflozin OR tofogliflozin) AND TITLE-ABSTRACT-INDEX TERM (RCT* OR random*)) |
| ClinicalTrials.gov | Canagliflozin OR dapagliflozin OR empagliflozin OR ertugliflozin OR ipragliflozin OR luseogliflozin OR sotagliflozin OR remogliflozin OR sergliflozin OR tofogliflozin |

1. MEDLINE: September 18, 2017

| Search | Query | Items found |
| --- | --- | --- |
| #1 | Sodium-glucose co-transporter [Title/Abstract/Text Word] | 584 |
| #2 | SGLT2 [Title/Abstract/Text Word] OR SGLT-2 [Title/Abstract/Text Word] OR SGLT 2 [Title/Abstract/Text Word]) | 1,592 |
| #3 | (Canagliflozin [Title/Abstract/Text Word] OR Invokana [Title/Abstract/Text Word]) | 497 |
| #4 | (Dapagliflozin [Title/Abstract/Text Word] OR Farxiga [Title/Abstract/Text Word] OR Forxiga [Title/Abstract/Text Word]) | 537 |
| #5 | (Empagliflozin [Title/Abstract/Text Word] OR Jardiance [Title/Abstract/Text Word]) | 542 |
| #6 | Ertugliflozin [Title/Abstract/Text Word] | 12 |
| #7 | (Ipragliflozin [Title/Abstract/Text Word] OR Suglat [Title/Abstract/Text Word]) | 107 |
| #8 | (Luseogliflozin [Title/Abstract/Text Word] OR Lusefi [Title/Abstract/Text Word]) | 63 |
| #9 | (Sotagliflozin [Title/Abstract/Text Word] OR LX4211 [Title/Abstract/Text Word]) | 19 |
| #10 | Remogliflozin [Title/Abstract/Text Word] | 18 |
| #11 | Sergliflozin [Title/Abstract/Text Word] | 14 |
| #12 | (Tofogliflozin [Title/Abstract/Text Word] OR Apleway [Title/Abstract/Text Word] OR Deberza [Title/Abstract/Text Word]) | 51 |
| #13 | OR #1-#12 | 2,320 |
| #14 | Random* [Title/Abstract/Text Word] | 928,396 |
| #15 | "Randomized Controlled Trial" [Publication Type] | 442,520 |
| #16 | (RCT [Title/Abstract/Text Word] or RCTs [Title/Abstract/Text Word]) | 32,266 |
| #17 | #14 OR #15 OR #16 | 1,046,506 |
| #18 | #13 AND #17 | 521 |

1. Embase: September 18, 2017

| Search | Query | Items found |
| --- | --- | --- |
| #1 | 'sodium-glucose cotransporter':ti,ab,kw | 1,026 |
| #2 | 'sglt2':ti,ab,kw OR 'sglt-2':ti,ab,kw OR 'sglt 2':ti,ab,kw | 2,961 |
| #3 | 'canagliflozin':ti,ab,kw OR 'invokana':ti,ab,kw | 901 |
| #4 | 'dapagliflozin':ti,ab,kw OR 'farxiga':ti,ab,kw OR 'forxiga':ti,ab,kw | 1,187 |
| #5 | 'empagliflozin':ti,ab,kw OR 'jardiance':ti,ab,kw | 973 |
| #6 | 'ertugliflozin':ti,ab,kw | 41 |
| #7 | 'ipragliflozin':ti,ab,kw OR 'suglat':ti,ab,kw | 179 |
| #8 | 'luseogliflozin':ti,ab,kw OR 'lusefi':ti,ab,kw | 116 |
| #9 | 'sotagliflozin':ti,ab,kw OR 'lx4211':ti,ab,kw | 61 |
| #10 | 'remogliflozin':ti,ab,kw | 31 |
| #11 | 'sergliflozin':ti,ab,kw | 19 |
| #12 | 'tofogliflozin':ti,ab,kw OR 'apleway':ti,ab,kw OR 'deberza':ti,ab,kw | 86 |
| #13 | OR #1-#12 | 4,275 |
| #14 | 'random*':ti,ab,kw | 1,219,216 |
| #15 | 'randomized controlled trial'/de | 464,254 |
| #16 | 'rct':ti,ab,kw OR 'rcts':ti,ab,kw | 50,090 |
| #17 | #14 OR #15 OR #16 | 1,321,849 |
| #18 | #13 AND #17 | 1,174 |

1. Cochrane Central Register of Controlled Trials: September 18, 2017

| Search | Query | Items found |
| --- | --- | --- |
| #1 | 'sodium glucose co-transporter':ti,ab,kw | 220 |
| #2 | 'sglt2':ti,ab,kw OR 'sglt-2':ti,ab,kw OR 'sglt 2':ti,ab,kw | 345 |
| #3 | 'canagliflozin':ti,ab,kw OR 'invokana':ti,ab,kw | 204 |
| #4 | 'dapagliflozin':ti,ab,kw OR 'farxiga':ti,ab,kw OR 'forxiga':ti,ab,kw | 279 |
| #5 | 'empagliflozin':ti,ab,kw OR 'jardiance':ti,ab,kw | 259 |
| #6 | 'ertugliflozin':ti,ab,kw | 16 |
| #7 | 'ipragliflozin':ti,ab,kw OR 'suglat':ti,ab,kw | 41 |
| #8 | 'luseogliflozin':ti,ab,kw OR 'lusefi':ti,ab,kw | 23 |
| #9 | 'sotagliflozin':ti,ab,kw OR 'lx4211':ti,ab,kw | 22 |
| #10 | 'remogliflozin':ti,ab,kw | 13 |
| #11 | 'sergliflozin':ti,ab,kw | 2 |
| #12 | 'tofogliflozin':ti,ab,kw OR 'apleway':ti,ab,kw OR 'deberza':ti,ab,kw | 11 |
| #13 | OR #1-#12 | 863 |
| #14 | 'random*':ti,ab,kw | 626,383 |
| #15 | 'randomized controlled trial':pt | 430,902 |
| #16 | 'rct':ti,ab,kw OR 'rcts':ti,ab,kw | 17,765 |
| #17 | #14 OR #15 OR #16 | 723,703 |
| #18 | #13 AND #17 | 722 |

**Supplementary Table 1.** Characteristics of included studies in the systematic review and meta-analysis

| Author | Year | Intervention | Control | Background antidiabetic drugs | Length of follow-up,  weeks | Number of participants | Mean age, years | Mean duration of diabetes,  years | Baseline eGFR,  mL/min/1.73 m^2^ | History of cardiovascular disease, *n* (%) | History of heart failure,  *n* (%) | Chronic kidney disease^a^,  *n* (%) |
| --- | --- | --- | --- | --- | --- | --- | --- | --- | --- | --- | --- | --- |
| Bailey^81^ | 2015 | Dapagliflozin 10 mg | Placebo | None | 102 | 145 | 52.2 | Intervention 2.1; Control 2.3 | SCr < 133 µmol/L (men), < 124 µmol/L (women) | N/A | N/A | N/A |
| Barnett^35^ | 2014 | Empagliflozin 10/25 mg | Placebo | OADs ± insulin ± GLP-1 RA | 52 | 741 | 63.9 | N/A | ≥ 15 | N/A | N/A | 448/738  (60.7%) |
| Bolinder^46^ | 2014 | Dapagliflozin 10 mg | Placebo | Metformin | 102 | 182 | 60.7 | Intervention 5.5; Control 6.0 | ≥ 60 | 47/180  (26.1%) | N/A | 6/180  (3.3%) |
| Cefalu^39^ | 2015 | Dapagliflozin 10 mg | Placebo | OADs (except TZD) ± insulin | 52 | 922 | 62.9 | N/A | ≥ 60 | 909/914  (99.5%) | N/A | N/A |
| Cherney^7,b^ | 2016 | Empagliflozin 10/25 mg | Placebo |  | 24 | 851 | 59.2 | N/A | ≥ 30 | N/A | N/A | 315/851  (37.0%) |
| Forst^49^ | 2014 | Canagliflozin 100/300 mg | Placebo | Metformin + TZD | 52 | 344 | 57.3 | 10.5 | ≥ 55 | N/A | N/A | N/A |
| Häring^32^ | 2013 | Empagliflozin 10/25 mg | Placebo | Metformin + SU | 24 | 669 | 57.1 | N/A | ≥ 30 | N/A | N/A | N/A |
| Häring^33^ | 2014 | Empagliflozin 10/25 mg | Placebo | Metformin | 24 | 638 | 55.7 | N/A | ≥ 30 | N/A | N/A | N/A |
| Heerspink^59^ | 2016 | Dapagliflozin 10 mg | Placebo | OADs ± insulin | 12 | 356 | 55.0 | Intervention 8.6; Control 8.3 | ≥ 60 | N/A | N/A | N/A |
| Inagaki^82^ | 2014 | Canagliflozin 10 mg | Placebo | None | 24 | 183 | 58.0 | 5.4 | ≥ 50 | N/A | N/A | N/A |
| Januzzi^83^ | 2017 | Canagliflozin 100/300 mg | Placebo | None OR  OADs ± insulin ± GLP-1 RA | 104 | 666 | 63.7 | Intervention 10.3 (median); Control 10.0 (median) | ≥ 50 | N/A | N/A | N/A |
| Ji^84^ | 2014 | Dapagliflozin 10 mg | Placebo | None OR metformin | 24 | 256 | 51.4 | Intervention 1.67; Control 1.30 | SCr < 133 µmol/L (men), < 124 µmol/L (women) | N/A | N/A | N/A |
| Ji^43^ | 2015 | Canagliflozin 100/300 mg | Placebo | Metformin ± SU | 18 | 678 | 56.2 | Intervention 6.8/6.9;  Control 6.4 | ≥ 60 | N/A | N/A | N/A |
| Kaku^85^ | 2014 | Tofogliflozin 20 mg | Placebo | None | 24 | 114 | 57.2 | Intervention 6.4; Control  6.0 | N/A | N/A | N/A | N/A |
| Kashiwagi^63^ | 2015 | Ipragliflozin 50 mg | Placebo | OADs | 52 | 165 | 64.4 | Intervention 9.5; Control 9.4 | ≥ 30, < 90 | N/A | N/A | 81/164  (49.4%) |
| Kohan^15^ | 2014 | Dapagliflozin 10 mg | Placebo | OADs ± insulin ± GLP-1 RA  ± pramlintide | 104 | 169 | 67.0 | Intervention 18.2; Control 15.7 | ≥ 30, ≤ 59 | N/A | N/A | 161/169  (95.3%) |
| Kovacs^34^ | 2014 | Empagliflozin 10/25 mg | Placebo | TZD ± metformin | 24 | 499 | 54.5 | N/A | ≥ 30 | N/A | N/A | N/A |
| Kovacs^61^ | 2015 | Empagliflozin 10/25 mg | Placebo | TZD ± metformin | 76 | 499 | 54.5 | N/A | ≥ 30 | N/A | N/A | N/A |
| Kosiborod^30,b^ | 2017 | Dapagliflozin 10 mg | Placebo |  | 52 | 340 | 64.2 | Intervention 13.5; Control 14.0 | ≥ 30, < 60 | 320/320  (100%) | 320/320  (100%) | N/A |
| Leiter^38^ | 2014 | Dapagliflozin 10 mg | Placebo | OADs (except TZD) ± insulin | 52 | 964 | 63.8 | Intervention 13.5; Control 13.0 | ≥ 60 | 959/962  (99.7%) | 152/962  (15.8%) | N/A |
| Lu^52^ | 2016 | Ipragliflozin 50 mg | Placebo | None | 24 | 171 | 53.7 | Intervention 6.5; Control 5.8 | ≥ 60 | N/A | N/A | N/A |
| Neal^9^ | 2017 | Canagliflozin 100/300 mg | Placebo | OADs ± insulin ± GLP-1 RA | 188 (mean) | 10,142 | 63.3 | 13.5 | > 30 | 7,324/10,142  (72.2%) | 1,461/10,142  (14.4%) | Intervention 36.4/1,000 PY; Control 49.3/1,000 PY |
| Perkovic^20,c^ | 2018 | Canagliflozin 100/300 mg | Placebo | OADs ± insulin ± GLP-1 RA | 188 (mean) | 10,142 | 63.3 | 13.5 | > 30 | 7,025/10,140  (69.3%) | 1,460/10,140  (14.4%) | 2039/10140  (20.1%) |
| Perkovic^22,c^ | 2019 | Canagliflozin 100 mg | Placebo | OADs ± insulin ± GLP-1 RA | 2.62 years (median) | 4,401 | 63.0 | 15.8 | ≥ 30, < 90 | 2,220/4,401  (50.4%) | 652/4,401  (14.8%) | 2,592/4,401  (58.9%) |
| Pollock^21,c^ | 2019 | Dapagliflozin 10 mg | Placebo | OADs ± insulin ± GLP-1 RA | 24 | 461 | 64.5 | Intervention 17.7; Control 17.6 | ≥ 20, ≤ 80 | 162/448  (36.1%) | N/A | N/A |
| Rodbard^51^ | 2016 | Canagliflozin 100-300 mg | Placebo | Metformin + sitagliptin | 26 | 218 | 57.5 | 9.9 | ≥ 60 | N/A | N/A | N/A |
| Rosenstock^62^ | 2015 | Empagliflozin 10/25 mg | Placebo | Insulin ± metformin ± SU | 78 | 494 | 58.8 | N/A | ≥ 30 | N/A | N/A | N/A |
| Søfteland^53^ | 2017 | Empagliflozin 10/25 mg | Placebo | Metformin + linagliptin | 24 | 333 | 55.2 | N/A | ≥ 60 | N/A | N/A | 5/327  (1.5%) |
| Strojek^86^ | 2014 | Dapagliflozin 10 mg | Placebo | SU | 48 | 597 | 59.8 | Intervention 7.2; Control 7.4 | ≥ 50 | 101/296  (34.1%) | N/A | N/A |
| Tikkanen^54^ | 2015 | Empagliflozin 10/25 mg | Placebo | None | 12 | 825 | 60.3 | N/A | ≥ 60 | N/A | N/A | N/A |
| Wanner^6^ | 2016 | Empagliflozin 10/25 mg | Placebo | OADs ± insulin ± GLP-1 RA | 3.1 years (mean) | 7,020 | 63.1 | N/A | ≥ 30 | 7,018/7,018  (100%) | 706/7,018  (10.1%) | 1,819/7,018  (25.9%) |
| Weber^40^ | 2016 | Dapagliflozin 10 mg | Placebo | OADs ± insulin | 12 | 588 | 56.5 | Intervention 7.7; Control 7.3 | ≥ 60 | 41/449  (9.1%) | N/A | N/A |
| Wilding^48^ | 2013 | Canagliflozin 100/300 mg | Placebo | Metformin ± SU | 52 | 469 | 56.8 | 9.6 | ≥ 55 | N/A | N/A | N/A |
| Wilding^65^ | 2014 | Dapagliflozin 10 mg | Placebo | OADs + insulin | 104 | 393 | 59.3 | Intervention 13.5; Control 14.2 | ≥ 50 | 75/387  (19.4%)^d^ | N/A | N/A |
| Yale^60^ | 2014 | Canagliflozin 100/300 mg | Placebo | OADs ± insulin ± GLP-1 RA | 52 | 272 | 68.5 | 16.3 | ≥ 30, < 50 | N/A | N/A | N/A |
| Cefalu^50^ | 2013 | Canagliflozin 100/300 mg | Glimepiride | Metformin | 52 | 1,452 | 56.2 | 5.0 (median) | ≥ 55 | N/A | N/A | N/A |
| DeFronzo^44^ | 2015 | (a) Empagli-flozin 10/25 mg OR (b) empagliflozin 10/25 mg + linagliptin | Linagliptin | Metformin | 52 | 686 | 56.4 | N/A | ≥ 60 | N/A | N/A | 18/674  (2.7%) |
| Frías^55^ | 2016 | (a) Dapagli-flozin 10 mg OR (b) dapagliflozin 10 mg + exenatide | Exenatide | Metformin | 28 | 695 | 54.3 | Intervention (a) 7.1, (b) 7.6; Control 7.4 | ≥ 60 | N/A | N/A | 25/680  (3.6%) |
| Hadjadj^57^ | 2016 | Empagliflozin 10/25 mg | Metformin | None | 24 | 680 | 52.6 |  | ≥ 60 | N/A | N/A | N/A |
| Heerspink^8^ | 2017 | Canagliflozin 100/300 mg | Glimepiride | Metformin | 104 | 1,452 | 56.2 | Intervention 6.5/6.7; Control 6.6 | ≥ 55 | N/A | N/A | N/A |
| Leiter^41^ | 2015 | Canagliflozin 100/300 mg | Glimepiride | Metformin | 104 | 1,452 | 56.2 | 6.6 | ≥ 55 | N/A | N/A | N/A |
| Lewin^56^ | 2015 | (a) Empagli-flozin 10/25 mg OR (b) empagliflozin 10/25 mg + linagliptin | Linagliptin | None | 52 | 677 | 54.6 | N/A | ≥ 60 | N/A | N/A | 17/667  (2.5%) |
| Nauck^47^ | 2011 | Dapagliflozin 10 mg | Glipizide | OADs | 52 | 816 | 58.5 | Intervention 6.0; Control 7.0 | ≥ 60 | 150/801  (18.7%) | N/A | 43/801  (5.4%) |
| Ridderstråle^45^ | 2014 | Empagliflozin 25 mg | Glimepiride | Metformin | 104 | 1,549 | 55.9 | N/A | ≥ 60 | N/A | N/A | 35/1,545  (2.3%) |
| Roden^31^ | 2013 | Empagliflozin 10/25 mg | (a) Placebo OR (b) Sitagliptin | None | 24 | 899 | 55.0 | N/A | ≥ 50 | N/A | N/A | N/A |
| Rosenstock^58^ | 2016 | (a) Canagli-flozin 100/300 mg + metformin OR (b) canagliflozin 100/300 mg | Metformin | None | 26 | 1,186 | 54.9 | 3.3 | ≥ 60 | N/A | N/A | N/A |
| Schernthaner^42^ | 2013 | Canagliflozin 300 mg | Saxagliptin | Metformin ± SU | 52 | 756 | 56.7 | 9.3 | ≥ 55 | N/A | N/A | N/A |
| Stenlöf^64^ | 2014 | Canagliflozin 100/300 mg | Placebo/  Sitagliptin | OAD mono-therapy (except TZD) OR Metformin + SU | 52 | 587 | 55.4 | 4.3 | ≥ 50 | N/A | N/A | N/A |

GLP-1 RA, glucagon-like peptide-1 receptor agonist; N/A, not applicable; SCr, serum creatinine; OADs, oral antidiabetic drugs; PY, patient-year; SU, sulfonylurea; TZD, thiazolidinedione. ^a^Chronic kidney disease is defined as eGFR < 60 mL/min/1.73 m^2^. ^b^These studies are pooled analyses of RCTs. ^c^Given the effect size and weight, we included two publications in our study which were published after the prespecified analysis. ^d^This includes patients with a history of coronary artery disease only.

**Supplementary Fig. 1.** Study quality and risk of bias assessment.

**Supplementary Fig. 2.** Subgroup analyses of microalbuminuria, macroalbuminuria, worsening nephropathy, and end-stage renal disease for canagliflozin, dapagliflozin, and empagliflozin. (A) Microalbuminuria. (B) Macroalbuminuria. (C) Worsening nephropathy. (D) End-stage renal disease. CI, confidence interval; RR, relative risk; W, weight.

(A)


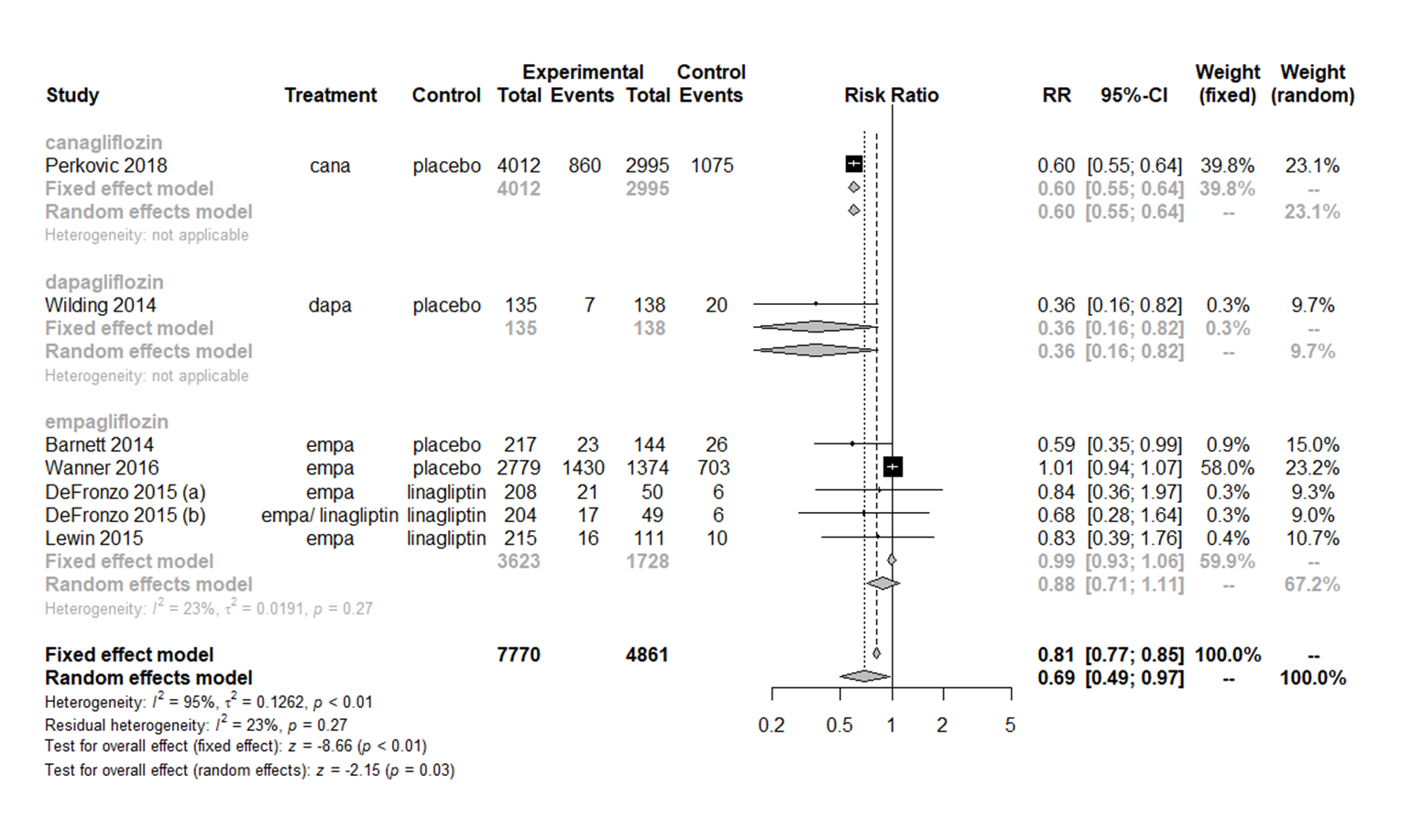


(B)


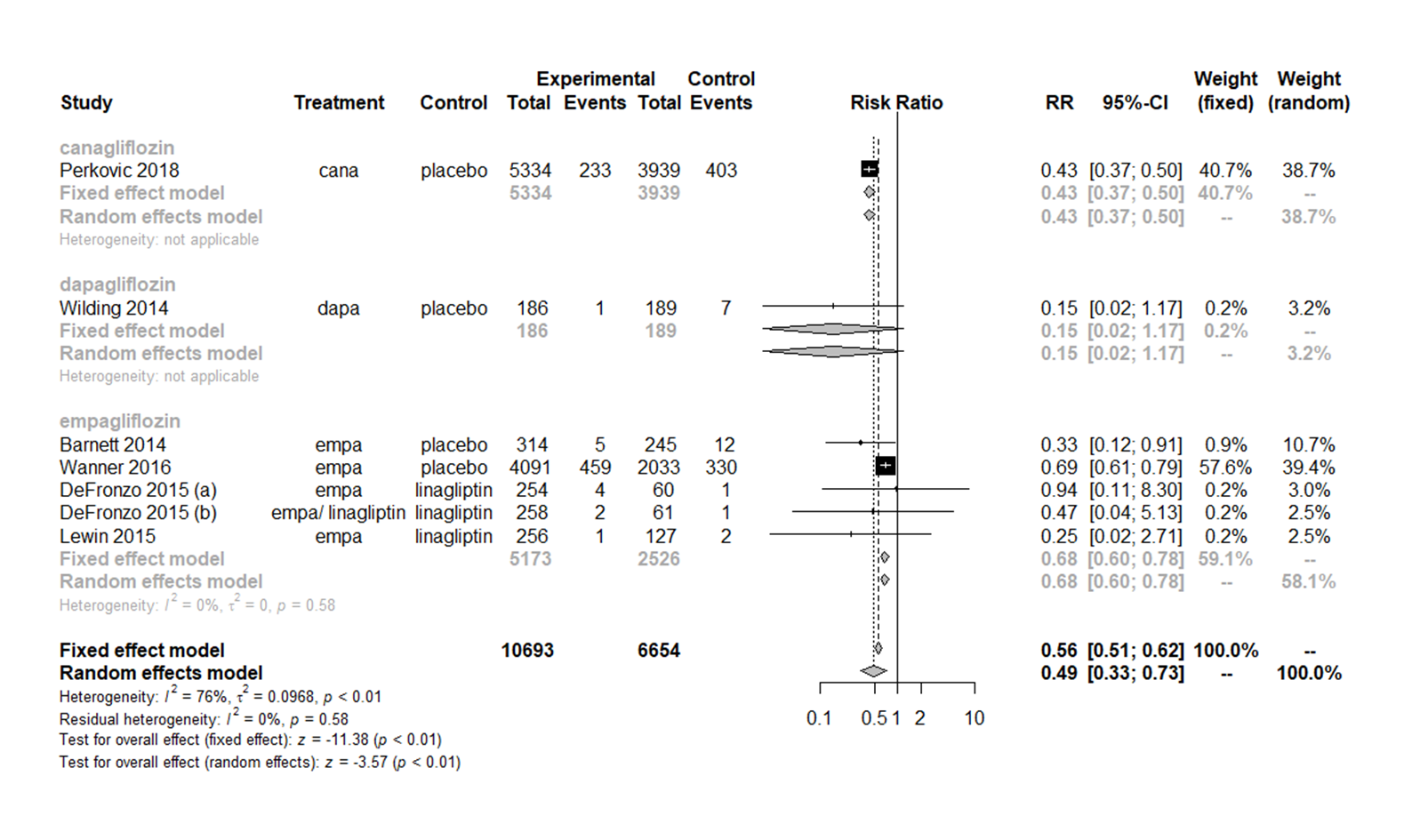


(C)


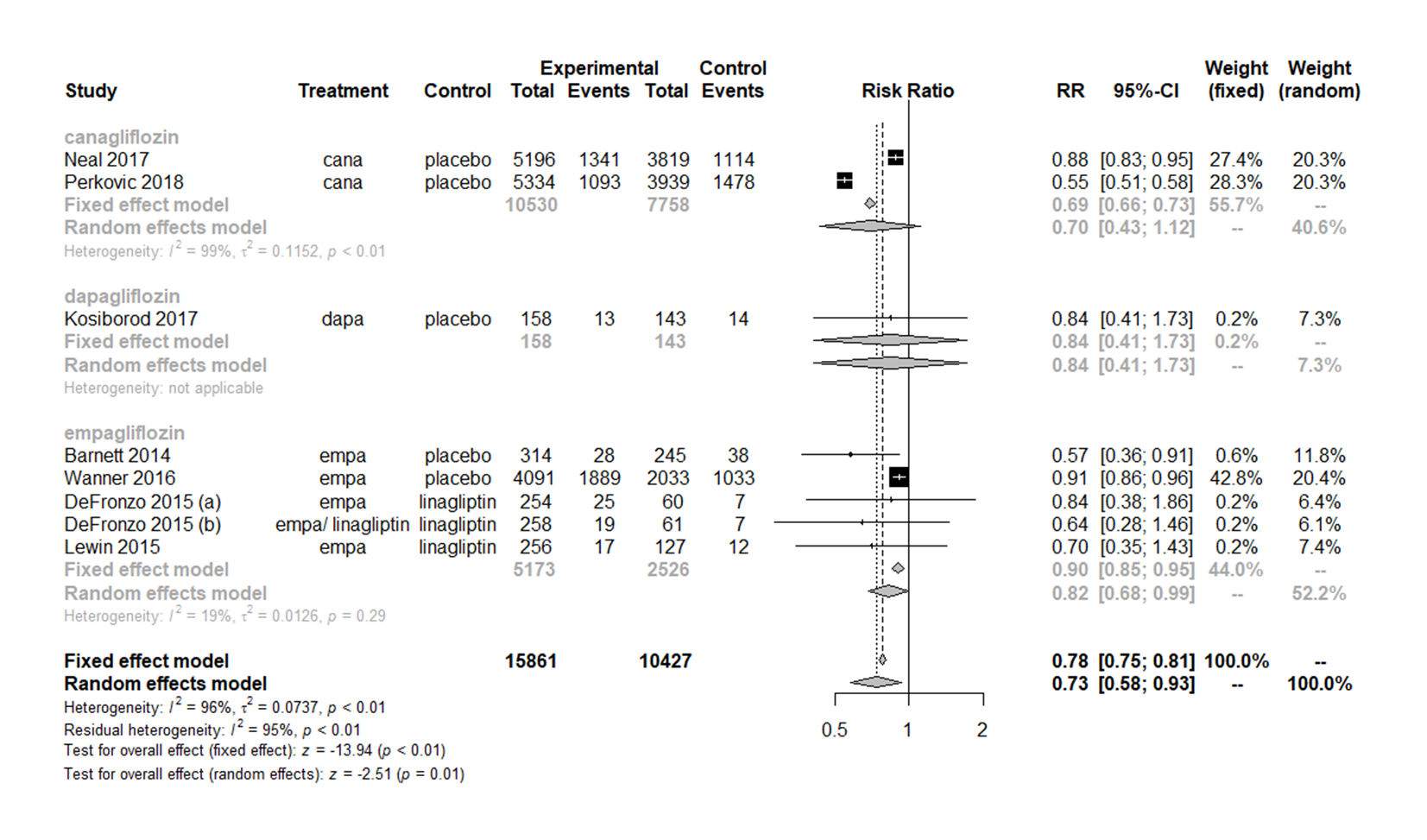


(D)


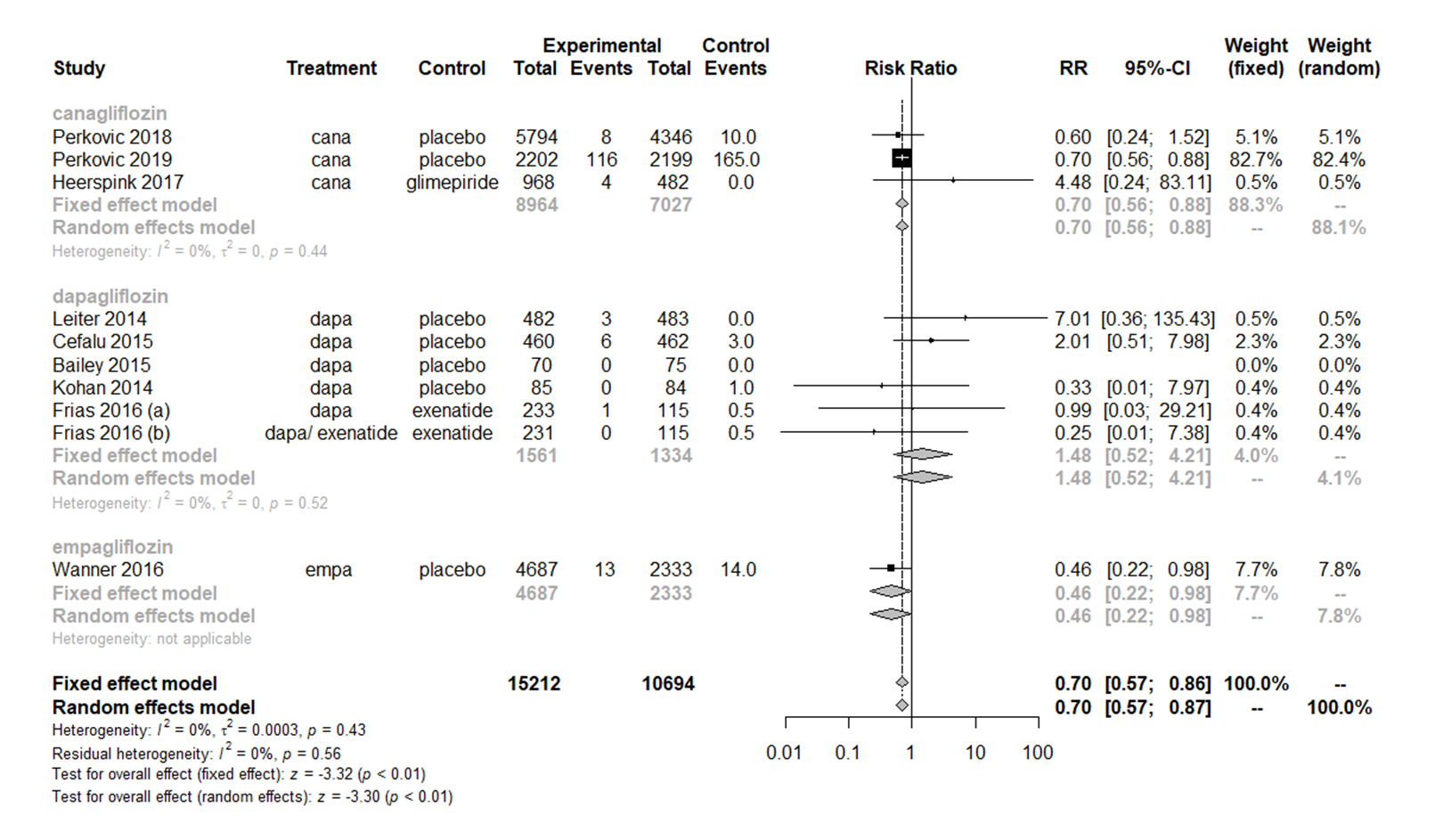


**Supplementary Fig. 3.** Funnel plots for changes in urine albumin-to-creatinine ratio and estimated glomerular filtration rate for sodium-glucose cotransporter 2 inhibitors versus placebo or other antidiabetic drugs. (A) Changes in urine albumin-to-creatinine ratio. (B) Changes in estimated glomerular filtration rate.

(A)


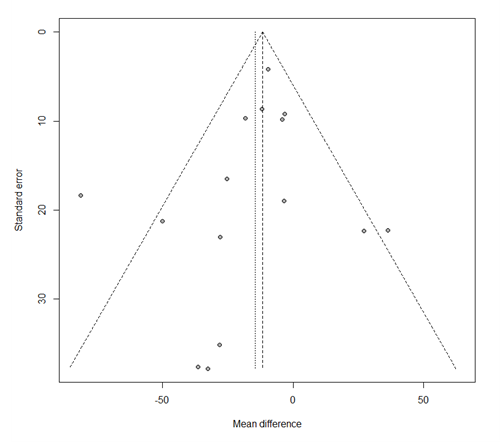


(B)


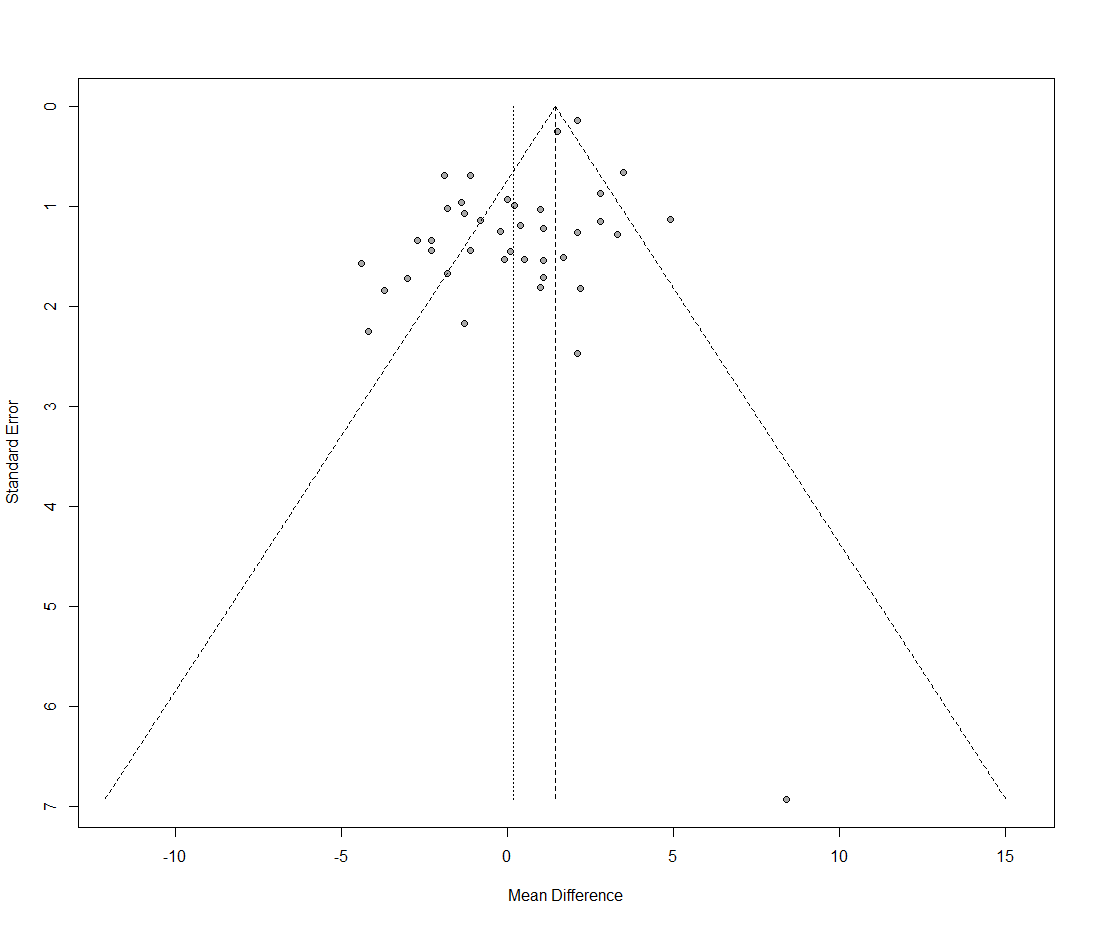


**Supplementary Fig. 4.** Funnel plots for the development of microalbuminuria, macroalbuminuria, worsening nephropathy, and end-stage renal disease for sodium-glucose cotransporter 2 inhibitors versus placebo or other antidiabetic drugs. (A) Microalbuminuria. (B) Macroalbuminuria. (C) Worsening nephropathy. (D) End-stage renal disease.

(A)


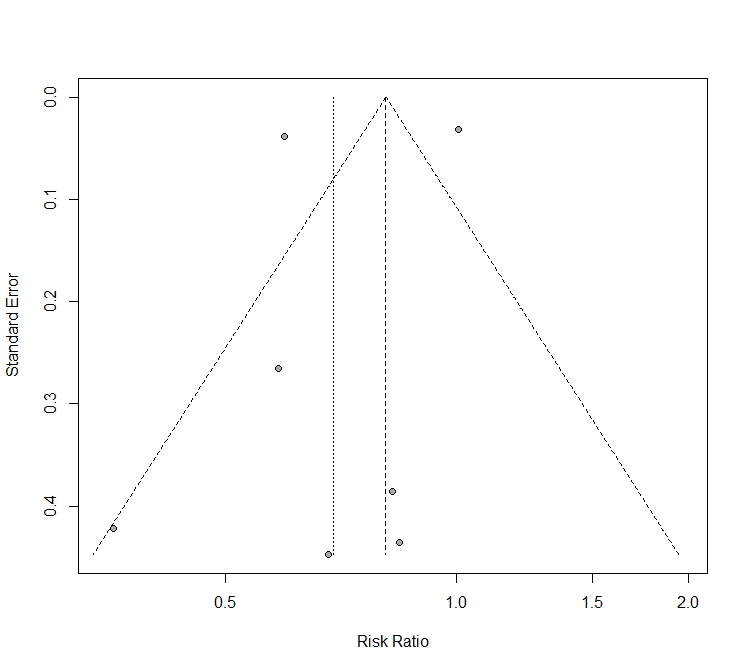


(B)


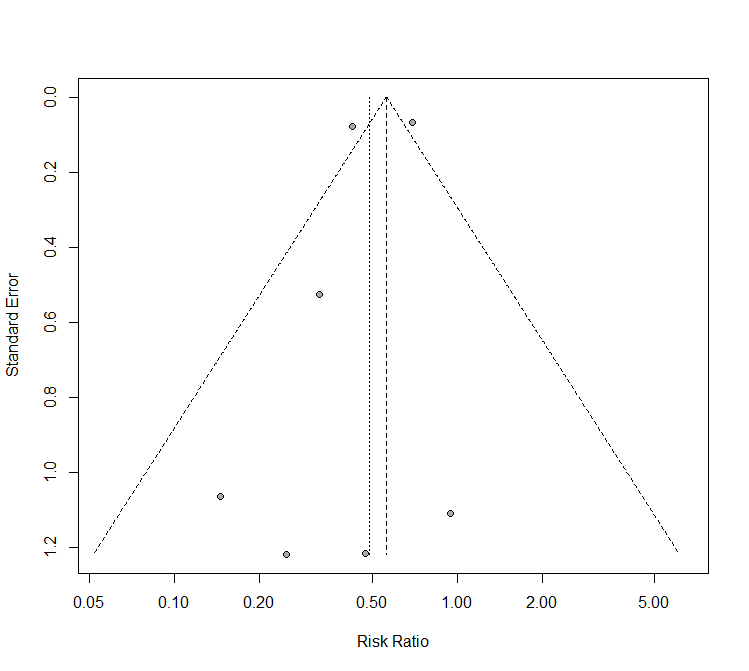


(C)


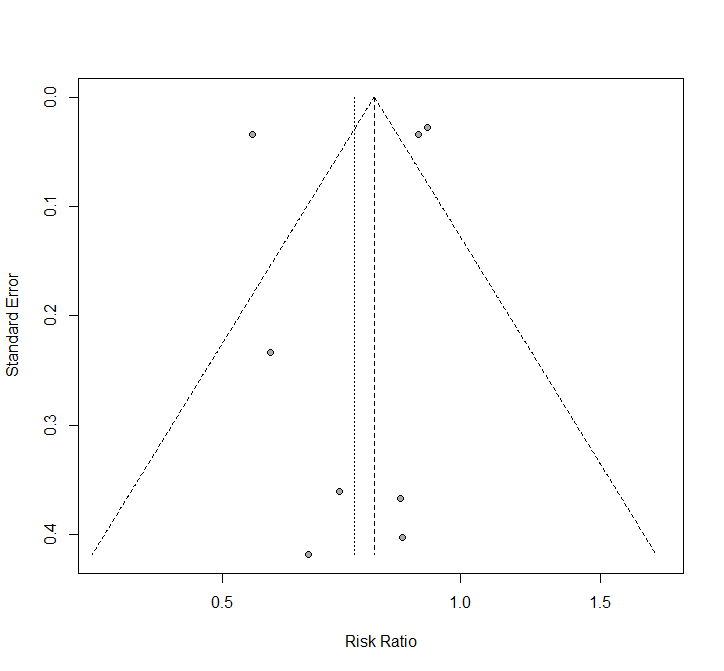


(D)


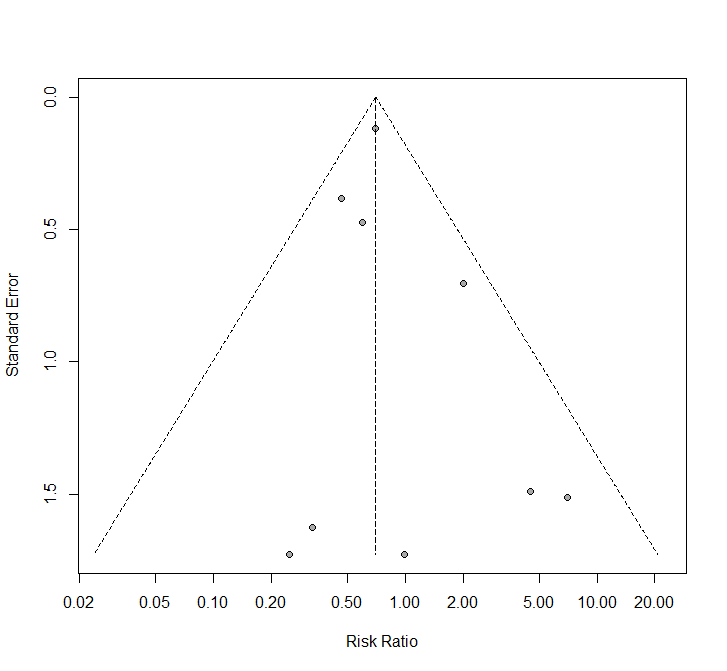


**References (continued)**

81 Bailey, C. J. *et al.* Efficacy and safety of dapagliflozin monotherapy in people with Type 2 diabetes: a randomized double-blind placebo-controlled 102-week trial. *Diabet Med* **32**, 531-541, doi:10.1111/dme.12624 (2015).

82 Inagaki, N. *et al.* Efficacy and safety of canagliflozin monotherapy in Japanese patients with type 2 diabetes inadequately controlled with diet and exercise: a 24-week, randomized, double-blind, placebo-controlled, Phase III study. *Expert Opin Pharmacother* **15**, 1501-1515, doi:10.1517/14656566.2014.935764 (2014).

83 Januzzi, J. L., Jr. *et al.* Effects of Canagliflozin on Cardiovascular Biomarkers in Older Adults With Type 2 Diabetes. *J Am Coll Cardiol* **70**, 704-712, doi:10.1016/j.jacc.2017.06.016 (2017).

84 Ji, L. *et al.* Dapagliflozin as monotherapy in drug-naive Asian patients with type 2 diabetes mellitus: a randomized, blinded, prospective phase III study. *Clin Ther* **36**, 84-100 e109, doi:10.1016/j.clinthera.2013.11.002 (2014).

85 Kaku, K. *et al.* Efficacy and safety of monotherapy with the novel sodium/glucose cotransporter-2 inhibitor tofogliflozin in Japanese patients with type 2 diabetes mellitus: a combined Phase 2 and 3 randomized, placebo-controlled, double-blind, parallel-group comparative study. *Cardiovasc Diabetol* **13**, 65, doi:10.1186/1475-2840-13-65 (2014).

86 Strojek, K. *et al.* Dapagliflozin added to glimepiride in patients with type 2 diabetes mellitus sustains glycemic control and weight loss over 48 weeks: a randomized, double-blind, parallel-group, placebo-controlled trial. *Diabetes Ther* **5**, 267-283, doi:10.1007/s13300-014-0072-0 (2014).
